# On the rapid cooling cast solidification microstructures of Mg–Ca–Zn alloys

**DOI:** 10.1007/s10853-025-11431-2

**Published:** 2025-11-08

**Authors:** Yanheng Xie, Magnus Anderson, Claire Utton, Dikai Guan, Matthew Murphy, Hector Basoalto

**Affiliations:** 1 School of Chemical, Materials and Biological Engineering, University of Sheffield, Sheffield, S1 3JD UK; 2 Thermo-Calc Software AB, Råsundavägen 18, 169 67 Solna, Sweden; 3 Department of Mechanical Engineering, School of Engineering, University of Southampton, Southampton, SO17 1BJ UK; 4 Luxfer MEL Technologies, Elektron Technology Centre, Lumns Lane, Manchester, M27 8LN UK

## Abstract

This work investigates the influence of Mg–Zn–Ca alloy compositions and rapid cooling conditions on microstructural evolution, with a focus on the formation and behaviour of intermetallic phases such as Mg_2_Ca, MgZn, and Ca_2_Mg_6_Zn_3_ during solidification. To achieve this, a combination of experimental characterisation and computational modelling was employed. The Scheil model, extended to ternary alloy systems, was used to simulate micro-segregation during solidification, while a multicomponent mean-field model was applied to predict solid-state phase transformations and the evolution of second-phase particles. CALPHAD-based thermodynamic calculations were integrated to refine the prediction of segregation pathways and phase distributions under non-equilibrium conditions. The model successfully differentiates solidification paths based on alloy composition, predicting that Mg–0.8Zn–0.2Ca (wt%) first forms Mg_2_Ca phase segregation, whereas Mg–6.8Zn–0.2Ca (wt%) primarily segregates MgZn. Experimental validation using SEM–EDS characterisation confirms these predictions. Finally, intermetallic phase formation diagrams under different solidification conditions are presented, providing insights into the control of intermetallic phase formation in Mg–Zn–Ca alloys.

**Supplementary Information:**

The online version contains supplementary material available at 10.1007/s10853-025-11431-2.

## Introduction

Magnesium (Mg) alloys, as the lightest structural metal, have great potential for use in automotive and aerospace applications [1–3]. However, the hexagonal crystalline structure of Mg limits slips systems at room temperature, leading to poor formability. Moreover, strong basal textures developed during processing contribute to pronounced yield asymmetry and low ductility [4–6], presenting a typical strength-formability trade-off for Mg alloys [7]. Rare earth (RE) elements are known to mitigate this issue by altering the texture of Mg alloys through the formation of RE-induced texture components, thereby improving ductility [8–11]. However, due to the limited availability and high cost of RE elements, calcium (Ca) has attracted attention as a potential substitute. Ca has a comparable atomic radius to RE elements and can induce similar texture-weakening effects in Mg alloys, but at a lower economic and environmental cost [12]. Weakened texture has been found in Mg–Zn–Ca ternary alloy [13–15]. Appropriate Zn additions refine grain size and contribute to solid solution and precipitation strengthening [16, 17], and the strength of Mg–Zn–Ca alloy is significantly higher than that of pure Mg [18–21].

Mg–Zn–Ca alloys have attracted significant attention in magnesium alloy research for biomedical devices due to their excellent biocompatibility. The second-phase formation in these alloys is strongly dependent on the Zn/Ca atomic ratio. Under equilibrium conditions, when the Zn/Ca atomic ratio > 1.2, typical phases include α-Mg, Mg_2_Ca, and Ca_2_Mg_6_Zn_3_ [22, 23]. Among these, Mg_2_Ca is coarse, brittle, and electrochemically active, with a lower electrode potential than α-Mg, leading to accelerated corrosion and reduced biomedical applicability [24, 25]. Alper and Ali [18] investigated mechanical performance as a function of Zn/Ca ratio and showed that ZX10 (Mg–0.9%Zn–0.2%Ca), which forms fewer Mg_2_Ca phases than ZX12 (Mg–0.9%Zn–1.5%Ca), exhibits higher tensile strength (127 MPa vs. 65 Mpa) and elongation (7.5% vs. 1.5%) despite having a larger average grain size (~ 844 μm vs. 635 μm). This is attributed to a reduction in Mg_2_Ca and the presence of fine, dispersed Ca_2_Mg_6_Zn_3_ precipitates that improve mechanical integrity [26]. However, the Mg_2_Ca phase has a high melting temperature of 715 °C, significantly above that of the Mg matrix, making it difficult to dissolve through heat treatment [27, 28]. Oh-ishi et al*.* [29] and Yang et al*.* [30] further noted that increasing Zn content can promote the formation of Mg–Zn binary phases such as MgZn, which, while beneficial for strength, lower the alloy’s melting point and may complicate thermal. In contrast, when Zn/Ca < 1.2, the stable phases include α-Mg, MgZn, and Ca_2_Mg_6_Zn_3_ [31]. The increase in MgZn delay the ageing response of Mg–Zn–Ca alloys [29], and grain boundary segregation of Ca_2_Mg_6_Zn_3_ may affect corrosion resistance [32]. To overcome these challenges, an effective strategy involves suppressing the formation of Mg_2_Ca and MgZn phases during solidification while achieving dispersed, uniform, and fine Ca_2_Mg_6_Zn_3_ phases within the α-Mg matrix through optimised compositional and heat treatment design. This approach holds promise for significantly improving the mechanical and corrosion resistance properties of Mg–Zn–Ca alloys.

The casting process for Mg alloys involves complex phenomena, including mass transport, heat transfer, and phase transformations, which dictate the final microstructure and properties. During solidification, liquid-to-solid transformations, heat transfer, and mass transport govern the formation of microstructural features, such as grain size and intermetallic phases in addition to solidification defects. These phase transformations are fundamentally driven by thermodynamic and kinetic principles. Chemical thermodynamics enables the determination of phase equilibria and driving forces for phase transformations, which are essential for understanding microstructural evolution. CALPHAD (calculation of phase diagrams) provides a robust framework for calculating phase equilibria, mobilities, and thermodynamic driving forces under equilibrium and non-equilibrium conditions, serving as a basis for modelling solidification and solid-state transformations [33–35]. For microstructure simulation, high-fidelity methods such as phase-field and cellular automaton (CA) models offer spatial resolution but are computationally intensive [36–38]. In contrast, simplified models such as the Scheil approximation are widely used to predict solute segregation and second-phase formation under rapid cooling, particularly in alloy design scenarios [39, 40]. Combining CALPHAD with Scheil and mean-field approaches enables efficient exploration of solidification behaviour and phase evolution, aiding alloy optimisation [41, 42].

Solidification modelling requires attention to solute transport and transformation mechanisms in both liquid and solid phases. Liquid-state diffusion is significantly faster than in the solid, affecting the extent of segregation and phase morphology. Post-solidification transformations such as particle growth, coarsening, and redistribution are driven by solid-state diffusion, and can be modelled using mean-field approaches. A mean-field model effectively describes particle dispersion evolution by employing precipitation kinetics. It captures Ostwald ripening behaviour, approximates precipitate morphology using simplified geometry, and uses mean values to represent chemical concentrations in particles, the matrix, and the particle–matrix interface, thereby simulating particle size distribution. The foundational mean-field description of particle coarsening, referred to as the LSW model, was derived by Greenwood [43], Lifshitz and Slyozov [44], and Wagner [45]. This model, developed for binary alloys and dilute particle dispersions, has been extended by numerous authors to multicomponent systems, incorporating nucleation, growth, and coarsening regimes [46–48]. The SFFK model is a multicomponent mean-field method that incorporates thermodynamic and kinetic parameters to describe complex precipitation behaviour in alloys. It has seen wide application in high-temperature alloy systems and is adapted here to Mg–Zn–Ca solid-state transformations [46].

The Scheil model commonly used in solidification studies provides a simplified but powerful framework to simulate phase evolution during solidification [39]. It assumes that diffusion in the solid phase is negligible and that the liquid phase is perfectly mixed, enabling rapid predictions of phase segregation and the formation of intermetallic compounds at different stages of solidification. While simplified, it offers reasonable predictions under non-equilibrium conditions. However, in ternary or multicomponent systems, its assumptions become limiting, especially regarding interaction effects between solutes, as it often oversimplifies segregation behaviour and fails to accurately capture phase interactions. To address this, we introduce a novel liquidus-minimising Scheil model tailored for ternary Mg–Zn–Ca systems. This method dynamically follows the steepest descent of the liquidus surface, offering improved representation of segregation behaviour compared to classical Scheil simulations. In this alloy system, the model helps clarify the competing formation pathways of Mg_2_Ca, MgZn, and Ca_2_Mg_6_Zn_3_, especially near grain boundaries where segregation is most pronounced. Combined with the SFFK mean-field model, liquidus-minimising Scheil approach provide a comprehensive prediction of how alloy composition and cooling rates affect phase evolution and microstructure.

This study aims to investigate how Mg–Zn–Ca alloy compositions and rapid cooling conditions affect microstructural evolution, with a specific focus on Mg_2_Ca, MgZn, and Ca_2_Mg_6_Zn_3_ phase formation. Through experimentation and by employing the liquidus-minimising Scheil model extended to ternary alloy systems, and integrating it with CALPHAD-based thermodynamic calculations, this study seeks to predict the segregation pathways and phase distributions under non-equilibrium solidification conditions. Furthermore, by clarifying the thermodynamic and kinetic mechanisms behind Mg_2_Ca formation and its interactions with other phases, this work contributes to the development of alloys with tailored microstructures and improved performance. The integrated approach aligns with integrated computational materials engineering (ICME) and the Materials 4.0 initiative [49] highlighting the predictive power of such models for microstructure–property design in lightweight alloy systems.

## Materials and methods

### Methodology

To investigate and control the formation of the Mg_2_Ca phase in Mg–Zn–Ca alloys, a combined experimental and computational approach was adopted to clarify the mechanisms of second-phase precipitation in rapidly solidified microstructures. Rapid cooling leads to solute enrichment in the remaining liquid, which influences the nucleation and growth of intermetallic compounds. Two hypotheses are considered to describe second-phase formation:

- Hypothesis 1: The liquid alloy solidifies directly into both the Mg matrix and second-phase intermetallic compounds simultaneously.
- Hypothesis 2: The liquid first solidifies into a supersaturated Mg matrix, and then the second-phase particles precipitate from the Mg matrix.

**Figure 1:**
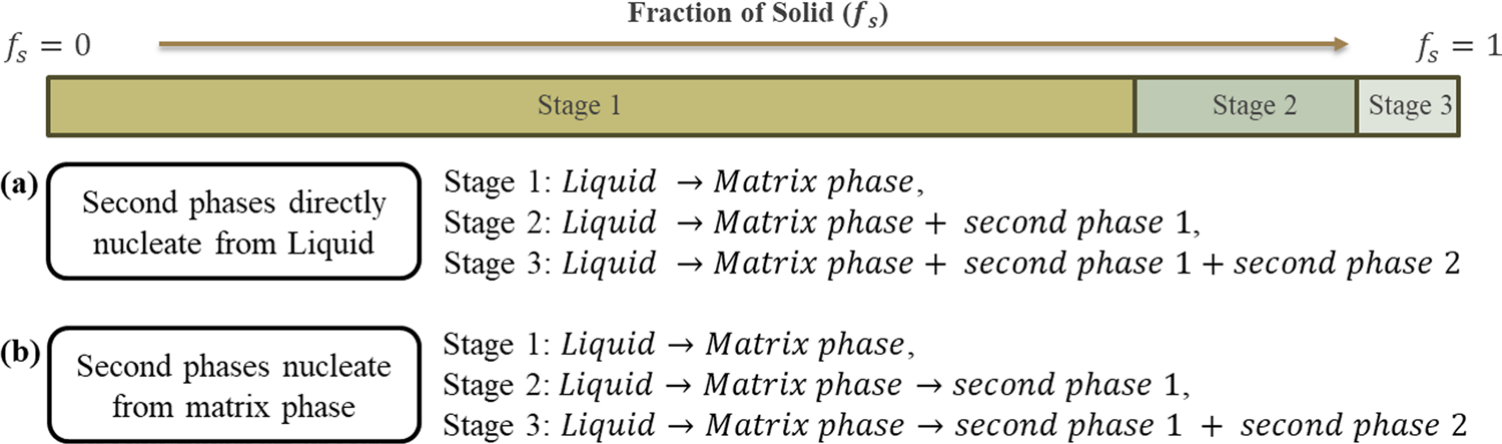
Hypothesis of precipitation sequences by **a** Scheil and **b** SFFK mean-field model of Mg–Zn–Ca alloys

Experiments have been performed to characterise the cast microstructures of ZX10 and ZX70. The Scheil model and the mean-field SFFK model are used to represent these two mechanisms, respectively, and their predictions are evaluated based on the experimental results. Figure 1 illustrates both precipitation sequences. While the Scheil model captures solute partitioning during solidification (Hypothesis 1), the SFFK model accounts for solid-state diffusion and coarsening after solidification (Hypothesis 2). In practice, both mechanisms may operate concurrently. The second-phase particles may nucleate during solidification, and then grow during the cool to room temperature through solid-state diffusion. This dual-model framework allows for a more comprehensive interpretation of phase formation in Mg–Zn–Ca alloys and aims to bridge theoretical predictions with observed microstructural features.

### Preparation of Mg alloys

**Figure 2:**
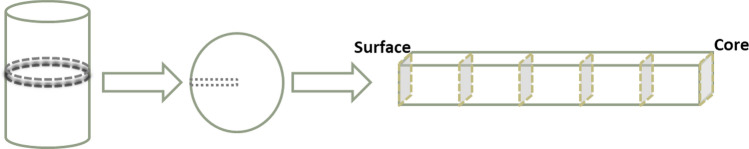
Schematic diagram of the cut area of the ZX10 and ZX70 samples

The as-cast Mg–0.8Zn–0.2Ca (ZX10) and Mg–6.8Zn–0.2Ca alloys (ZX70) were provided by Luxfer MEL Technology (UK). The molten alloys were poured into cylindrical steel mould equipped with a water-cooling system to achieve rapid solidification. The mould water temperature was maintained at ~ 25 °C, and the pouring temperature was approximately 750 °C. Heat transfer simulations estimated the average cooling rate to be 2 K/s in Sect. 3.2. The samples were removed from the mould approximately 10 min after solidification and subsequently cylindrical surfaces were polished to produce cylindrical billets with a diameter of 75 mm. A disc-shaped specimen was extracted from the centre of each billet, and a 37.5 × 3 × 3 mm sample was cut from the centre to the surface of the disc. This sample was then divided into five equal-length specimens, as illustrated in Fig. 2. The bulk compositions of both ZX10 and ZX70 alloys were measured using inductively coupled plasma optical emission spectroscopy (ICP-OES), multiple local compositions were obtained via SEM–EDS. The observed variation between ICP-OES and SEM–EDS values reflects local solute inhomogeneity, especially near grain boundaries. The measured compositions are presented in Table 1.

**Table 1:** Chemical composition of as-received Mg–Zn–Ca alloy

Alloy	Mg (wt%)	Zn (wt%)	Ca (wt%)	Measured by
ZX10	Bal	0.8	0.2	ICP-OES
ZX70	Bal	6.8	0.2	ICP-OES
ZX10	Bal	0.8–1.2	0.2–0.5	SEM–EDS
ZX70	Bal	6.4–7.2	0.2–0.3	SEM–EDS

### Microstructure characterisation

Samples for SEM and EDS were prepared by mechanical grinding and polishing. Samples were firstly ground by silicon carbide grinding papers and then polished by 1 µm, 0.25 µm alcohol-based diamond suspension. The final polishing was done by 40 nm colloidal silica suspension. SEM and EDS were operated on a Inspect FEI F50 SEM equipped with an Oxford Instruments AZtec Xmax-170 detector. The accelerating voltage for SEM image acquirement and EDS scanning was both at 20 kV. EDS data were collected and analysed by the Oxford Instruments Aztec software.

### Scheil solidification model

The Scheil model is based on three fundamental assumptions:

- The solidified phase is considered to be in a “frozen” state, meaning that reverse diffusion in the solid phase is neglected.
- Diffusion in the liquid phase is assumed to be sufficiently rapid to maintain a homogeneous composition in the liquid.
- The liquid and solid phases are in phase equilibrium at the local interface.

The classical Scheil equation provides a reasonable approximation of the solute distribution in the solid phase and the proportion of the eutectic composition formed during solidification. These relationships are expressed through the following equations [39]:

1 $${C}_{L}={C}_{0}{\left({f}_{L}\right)}^{k-1}$$

2 $${C}_{s}={kC}_{0}{\left(1-{f}_{s}\right)}^{k-1}$$

3 $$k=\frac{{C}_{s}}{{C}_{L}}$$

where ${C}_{L}$ and ${C}_{s}$ represent the solute concentrations in the liquid and solid phases, respectively, ${C}_{0}$ is the nominal composition, ${f}_{L}$ is the liquid fraction, ${f}_{s}$ is the solid fraction, and solute segregation is governed by the partition coefficient $k$. In classical Scheil solidification, the concentration of the liquid phase increases progressively during solidification due to compositional segregation, which in turn gradually alters the composition of the solidified phase. In a binary eutectic system, the solidification path follows the liquidus line as segregation continues until the eutectic point is reached, leading to the formation of a second-phase intermetallic compound. The solid fraction at any stage of solidification can be calculated using the lever rule through the following equation [50]:

4 $$\begin{aligned} f_{s} \left( i \right) & = \left| {\frac{{w_{b} \left( i \right) - w_{b} \left( 0 \right)}}{{w_{b} \left( i \right)}}} \right| \\ & w_{b} \left( 0 \right) < w_{b} \left( i \right) < w_{b} \left( {end} \right) \\ \end{aligned}$$

where the initial composition of $B$ is ${w}_{b}(0)$, the composition at the eutectic point is ${w}_{b}(end)$, the real-time composition during solidification is ${w}_{b}(i)$, and the solid fraction ${f}_{s}\left(i\right)$ at the $i$th composition. In ternary systems, the Scheil solidification process becomes significantly more complex due to the interaction between three solutes. The work of Chen et al*.* [40] and Boettinger et al*.* [51] demonstrated a ternary eutectic solidification model and a Scheil solidification path for ternary alloys, where the newly formed solid phase in local equilibrium would be in mass balance with the progressively segregating liquid phase due to the composition of the solid phase not evolved during the solidification process. The assumptions of the Scheil model remain unchanged, but in a ternary alloy system, the solidification path must account for interactions among three components. Here, the binary liquidus line expands into a liquidus surface, and the solidification path follows the steepest gradient as the liquid composition evolves. The segregation of two solutes occurs simultaneously, with their concentrations in the liquid phase being progressively partitioned into the solid. As the path approaches the boundaries of phase stability, it transitions along monovariant lines, capturing critical solidification behaviours [40]. The process concludes at the ternary eutectic point, where three phases solidify simultaneously under thermodynamic equilibrium. The solid fraction of solidification in ternary system can be calculated using the lever rule through the following equation:

5 $$\begin{aligned} f_{s} \left( i \right) & = \left| {\frac{{\sqrt {\left( {w_{b} \left( i \right) - w_{b} \left( 0 \right)} \right)^{2} + \left( {w_{c} \left( i \right) - w_{c} \left( 0 \right)} \right)^{2} } }}{{\sqrt {w_{b} \left( i \right)^{2} + w_{c} \left( i \right)^{2} } }}} \right| \\ & w_{b} \left( 0 \right) < w_{b} \left( i \right) < w_{b} \left( {end} \right) \\ & w_{c} \left( 0 \right) < w_{c} \left( i \right) < w_{c} \left( {end} \right) \\ \end{aligned}$$

In a ternary phase diagram, the phase fractions are determined using the area ratio method, where the fraction of each phase is proportional to the area of the opposite sub-triangle within the tie-triangle. This approach extends the binary lever rule and allows phase fractions to be approximated based on tie-triangle sub-areas in ternary diagrams.

### Mean-field SFFK model

The mean-field SFFK model is used to capture the precipitation kinetics of all intermetallic phases in Mg–Zn–Ca alloys. The composition is predicted from a Scheil calculation of chemical segregation during liquid solidification. The particle shape is assumed to be spherical, and the growth rate is considered to be a function of particle size and composition. The particle size distribution is described by a distribution function $F(R,t)$, which represents the number of particles with radius varying between the closed limit of $R$ and $R+dR$ at a specific time t and in per unit volume. Moments of the distribution function provide the following key statistical information regarding the dispersion.

6 $$\begin{aligned} & N\left( t \right) = \mathop \smallint \limits_{0}^{\infty } F\left( {R,t} \right) dR \\ & \overline{R}\left( t \right) = \mathop \smallint \limits_{0}^{\infty } RF\left( {R,t} \right) dR/\mathop \smallint \limits_{0}^{\infty } F\left( {R,t} \right) {\text{d}}R \\ & \phi \left( t \right) = \frac{4\pi }{3}\mathop \smallint \limits_{0}^{\infty } R^{3} F\left( {R,t} \right) {\text{d}}R \\ \end{aligned}$$

where $N\left(t\right)$ is particle concentration, $\overline{R }\left(t\right)$ is mean particle radius, and $\phi \left(t\right)$ is particle volume fraction. The evolution of the particle distribution is determined by solving the continuity equation,

7 $$\frac{\partial F(R,t)}{\partial t}+\frac{\partial \left[F\left(R,t\right)V\left(R,t\right)\right]}{\partial R}={\mathcal{F}}^{+}\left(R,t\right)-{\mathcal{F}}^{-}\left(R,t\right)$$

where the particle growth rate is given by $V\left(R,t\right)$, and ${\mathcal{F}}^{+}\left(R,t\right)$ and ${\mathcal{F}}^{-}\left(R,t\right)$ refer to source and sink terms, which representing nucleation and dissolution respectively in this model. The particle growth rate for spherical particles which describing Ostwald ripening kinetics is [52],

8 $$V\left(R,t\right) = \frac{A\left(t\right)}{R(t)} \left(\frac{1}{{R}_{c}\left(t\right)} - \frac{1}{R(t)} \right)z\left(R,t\right)$$

9 $$z\left(R,t\right)=1+R(t)\sqrt{4\pi {N}_{v}\left(t\right)\overline{R }\left(t\right)}$$

where the $A\left(t\right)$ represents the effective diffusion rate at the particle interface, and ${R}_{c}\left(t\right)$ is the critical particle radius. Particles smaller than ${R}_{c}\left(t\right)$ will dissolve and those bigger than ${R}_{c}\left(t\right)$ will grow. Term $z\left(R,t\right)$ is a correction factor accounting for non-dilute precipitate dispersions. The expression given is determined by Marqusee and Ross [52] for accounting the overlap of diffusion fields between neighbouring particles to accelerating particle growth kinetics. In the SFFK model, term $A\left(t\right)$ and ${R}_{c}\left(t\right)$ are listed below:

10 $$A\left(t\right)=\frac{2\gamma }{{R}_{g}T}\theta$$

11 $$\theta ={\left[{\sum }_{i=1}^{n}\frac{{\left({c}_{ki}-{c}_{0i}\right)}^{2}}{{c}_{0i}{D}_{0i}}\right]}^{-1}$$

12 $${R}_{c}\left(t\right)=\frac{2\gamma }{\Delta {G}_{c}}$$

13 $$\Delta {G}_{c}=- {\sum }_{i=1}^{n}{c}_{ki} \left({\mu }_{ki}-{\mu }_{0i}\right)$$

where $\gamma$ is interfacial energy, $\Delta {G}_{c}$ is chemical driving force, and ${c}_{ki}$ and ${c}_{0i}$ is molar concentrations of the ith alloying element in the particle and matrix phases, respectively. ${\mu }_{ki}$ and ${\mu }_{0i}$ refer to the chemical potentials of the precipitate and matrix phases considering the ith alloying element in an alloy with $n$ many alloying elements. The diffusivity of the $i$th alloying element within the matrix is given by ${D}_{0i}$.

The classical nucleation theory is given to describe the transient nucleation rate for homogenous nucleation of spherical particles [34],

14 $${\mathcal{F}}^{+}\left(R,t\right)=Z{\beta }^{*}\left(t\right){N}_{c}\left(R,t\right)\text{exp}\left(\frac{-\Delta {G}^{*}}{{k}_{b}T}\right){P}_{inc}$$

where the $Z$ term is the Zeldovich parameter, ${\beta }^{*}\left(t\right)$ is atomic attachment rate, ${N}_{c}\left(R,t\right)$ is nuclei radius distribution function, $\Delta {G}^{*}$ is energy barrier to nuclei formation, ${k}_{b}$ is Boltzmann constant, and ${P}_{inc}$ is nuclei incubation probability. The Zeldovich parameter is given by Jou et al*.* [53],

15 $$Z=\sqrt{\frac{{\Omega }^{2}\gamma }{4{\pi }^{2}{k}_{B}T{R}_{c}^{4}}}$$

where $\Omega$ is atomic volume, and ${k}_{b}$ is Boltzmann constant. The atomic attachment rate for a multicomponent is approximate by Svobda et al*.* [46],

16 $${\beta }^{*}=\frac{4\pi {R}_{c}^{2}}{{a}^{4}{V}_{m}} \theta$$

where $a$ is lattice parameter, and ${V}_{m}$ is molar volume. Jou et al*.* [53] also provide a Gaussian waveform to describe the distribution of nuclei concentration density. Anderson et al. [34] extended this to provide an estimate of the standard deviation based upon the Zeldovich factor descriptive of the gradient of the Gibbs free energy as a function of radius,

17 $$\begin{aligned} & N_{c} \left( {R,t} \right) = \frac{{N_{0} }}{{\delta \sqrt {2\pi } }}\exp \left( { - \frac{1}{2}\left( {\frac{{R - R_{c} \left( t \right)}}{\delta }} \right)^{2} } \right) \\ & N_{0} = \eta \frac{{3\left( {\phi_{eq} - \phi \left( t \right)} \right)}}{{4\pi R_{c}^{3} }} \\ & \delta = \left( {\frac{{3{\Omega }}}{{2\left( \pi \right)^{\frac{3}{2}} }} \frac{1}{Z} } \right)^{\frac{1}{3}} \\ \end{aligned}$$

where ${N}_{0}$ is the concentration of nuclei sites and $\delta$ is the variance of the nuclei size distribution, and $\eta$ refers to the fraction of active nucleation sites. For homogeneous nucleation, $\eta$ is unity. To approximate $\eta$ for heterogeneous nucleation, $\eta$ is given by the ratio of the available nucleation sites divided by the total number of nucleation sites within the volume of interest [35]. ${\phi }_{eq}$ is the equilibrium volume fraction of precipitates, $\Omega$ is atomic volume, and $Z$ is Zeldovich parameter. For spherical particles, the nucleation energy barrier is given by,

18 $$\Delta {G}^{*}=\frac{16\pi }{3}\frac{{\gamma }^{3}}{{\left(\Delta {G}_{c}\right)}^{2}}$$

The incubation probability ${P}_{inc}$ describes nucleation during a complex thermal cycle and indicates the likelihood of stable nuclei forming during transient nucleation, where ${P}_{inc}$ is defined as the ratio of the current nucleation concentration to the steady-state nucleation concentration.

19 $${P}_{inc}=\text{exp}\left(\frac{-\tau }{t}\right)$$

where $\tau$ is incubation time,

20 $$\tau =\frac{1}{2{\beta }^{*}{Z}^{2}}$$

If Eqs. (19), (14), (15) are substituted into Eq. (18), the incubation probability is given by,

21 $${P}_{inc}\left(t,T,\theta ,{R}_{c},\gamma \right) =\text{exp}\left(\frac{-\tau }{t}\right) =\text{exp}\left(-\frac{1}{t} \frac{{k}_{B}T{R}_{c}^{2}}{2\theta \gamma {a}^{2}}\right)$$

The temporal evolution of the incubation probability is given from Anderson et al*.* [34], and introduce a ${t}_{eq}$ as equivalent incubation time when ${0<P}_{inc}<1$.

22 $$\frac{{dP}_{inc}}{dt}=\frac{\tau }{{t}_{eq}} {P}_{inc} \left[\frac{1}{{t}_{eq}} +\left(\frac{1}{\theta } \frac{d\theta }{dT}-\frac{2}{{R}_{c}} \frac{dR}{dT}+\frac{1}{\gamma } \frac{d\gamma }{dT}-1\right) \frac{dT}{dt}\right]$$

23 $${t}_{eq}= -\frac{\tau }{\text{ln}\left({P}_{inc}\right)}$$

## Numerical implementation

The chemical potentials and diffusion rates required for this solidification model were obtained from the thermodynamic database TCMG6 and the mobility database MOBMG1 [54] in the commercial software Thermo-Calc 2024b [55]. A Fortran programme has been written to couple with the TQ-FORTRAN interface in Thermo-Calc software to capture the thermodynamic data of both Mg–0.8Zn–0.2Ca wt% (ZX10) and Mg–6.8Zn–0.2Ca wt% (ZX70) alloys during the simulation process.

### Solute segregation during solidification

The liquidus surface temperatures of Mg–Zn–Ca alloys in magnesium-rich corners were determined using the TQ-FORTRAN interface in the Thermo-Calc software. A simple finite difference numerical model was then constructed to calculate the liquidus-minimising Scheil solidification path for ternary alloy. During the collection of liquidus temperatures, the Zn content varied from 0 to 40 at.%, the Ca content ranged from 0 to 12 at.%, and the collection step size was 0.01 at.%. In total, 4.8 million nodes of phase equilibrium temperatures were collected.

**Figure 3:**
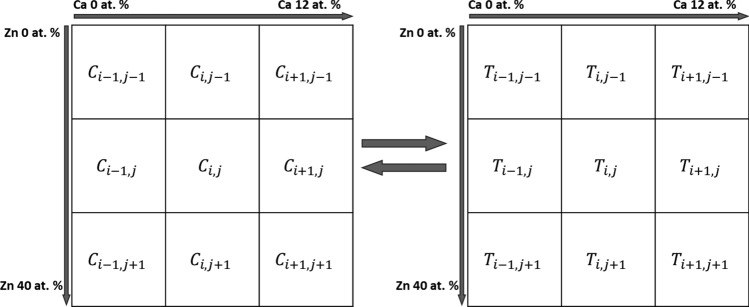
Liquidus-minimising Scheil model framework

As shown in Fig. 3, by importing these composition nodes and liquidus temperatures into the numerical model, the composition and liquidus diagram for the magnesium-rich corner of the Mg–Zn–Ca alloy system can be generated. The initial composition and corresponding liquidus temperature ${T}_{i,j}$ are assigned to the node ${C}_{i,j}$​, which serves as the starting point for the Scheil solidification path. The temperature difference $dT$ between ${T}_{i,j}$​ and its four neighbouring nodes is calculated to identify the neighbouring node with the lowest temperature, which determines the next point on the liquidus for the Scheil path. If two adjacent nodes have the same $dT$, the temperature value of the diagonal node between these two is used as the next point in the path. The composition and liquidus temperature at this new point become the updated ${C}_{i,j}$​ and ${T}_{i,j}$​, and the calculation is iteratively repeated to trace the full Scheil solidification path.

**Figure 4:**
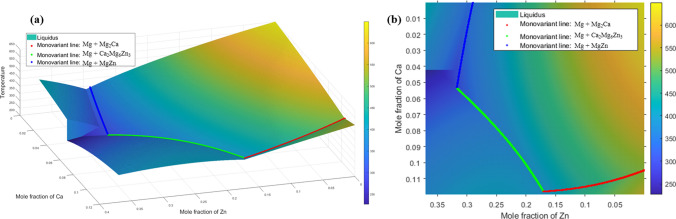
**a** 3D view and **b** 2D view liquidus projection of the Mg–Zn–Ca alloy system from TCMG6

The liquidus-minimising Scheil solidification path concludes when it reaches the boundary, defined as the monovariant line. The monovariant line can be calculated using thermodynamic equilibrium phase data obtained from the TCMG6 database via the TQ-FORTRAN interface. In the Mg–Zn–Ca ternary alloy system, the intersection of two monovariant lines defines the ternary eutectic point. The monovariant line is divided into $n$ nodes, with the composition ${C}_{i,j}$​ and liquidus temperature ${T}_{i,j}$​ of each node recorded. The liquidus surface temperature for the magnesium-rich corner of the Mg–Zn–Ca alloy system and the monovariant line are shown in Fig. 4. After the Scheil solidification path intersects the monovariant line, it continues along the gradient of decreasing temperature until it reaches the ternary eutectic point. All nodes encountered during this process are recorded and plotted as the solidification curve. Using the TCMG6 thermodynamic database, the thermodynamic parameters for each point along the solidification curve can be extracted, providing critical information such as the starting temperature, eutectic temperature, element segregation curves, and phase fractions during solidification. Video illustrating the solidification paths for ZX10 and ZX70 alloys are available in the supplementary materials (S1, S2).

### Cooling process

**Figure 5:**
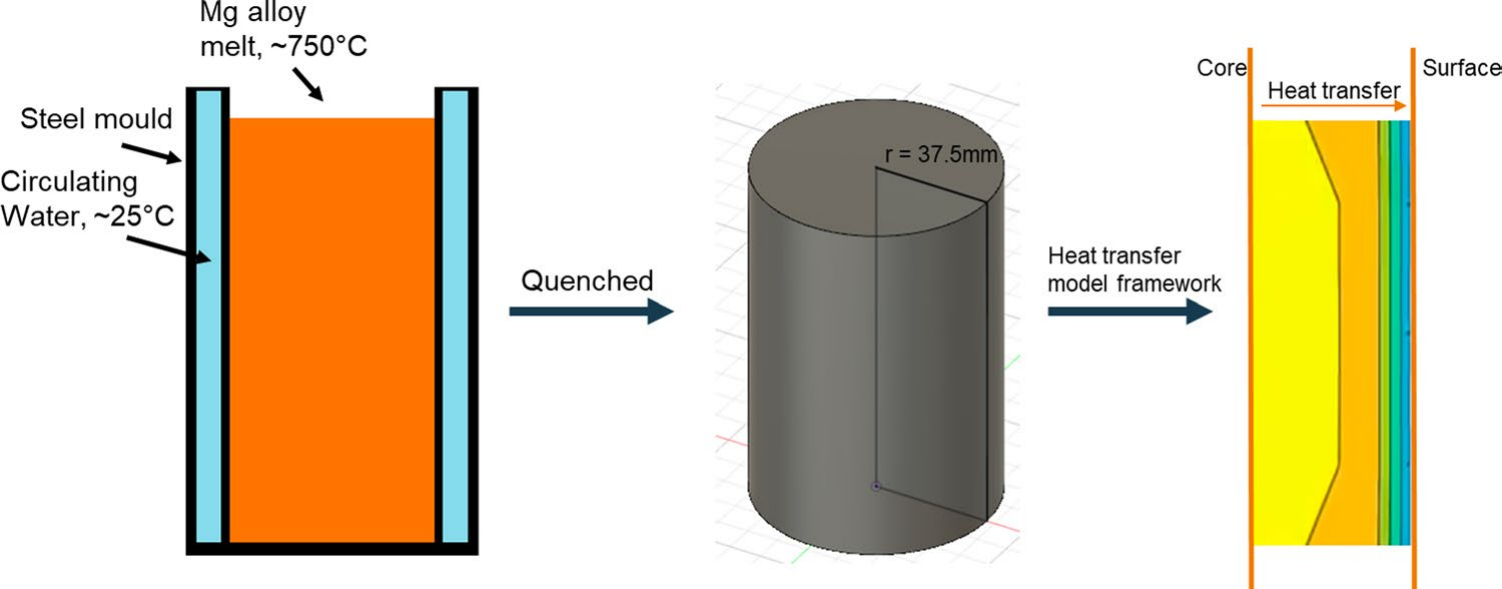
Sample rapid cooling process

**Figure 6:**
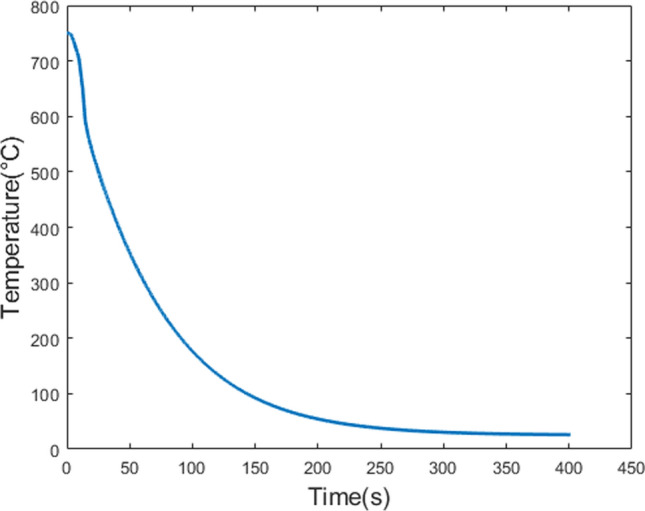
Approximate thermal history of ZX10 and ZX70 alloys cooling process

To replicate the thermal history of the rapid cooling process, heat transfer calculations under ideal conditions were conducted using the finite difference method. The casting solidifies progressively from the surface to the core, as illustrated in Fig. 5. The system was simplified as radial heat flow from the billet surface to its centre, with the geometry and orientation consistent with the experimental setup (Fig. 2). The parameters used for the one-dimensional heat transfer model are detailed in Table 2. The approximate thermal history results obtained from these calculations are presented in Fig. 6. It is important to note that this model describes the solidification process of a casting under ideal conditions. In practical production, surface shrinkage of the casting can create gaps between the billet and the mould, reducing heat transfer efficiency. As a result, the actual cooling time may be longer than predicted by this model. The model estimated cooling rates of 2 K/s for near-surface regions, consistent with rapid solidification conditions. These values align with typical water-cooled mould casting processes, such as chill casting or HPDC. The model also assumes cooling times from 5 to 120 min to observe solid-state phase changes under non-rapid cooling. To extend this framework to industrial practice, the modelling protocol can be adapted for use in commercial casting simulation software, enabling digital casting process optimisation.

**Table 2:** Model parameters for heat transfer calculation, where temperature is given by $T$ with units of Kelvin

Variable	Description	Value	Units	References
${k}_{L}$	Thermal conductivity	16.51 + 0.07 *$T$	W/m/K	Thermo-Calc
${k}_{\alpha }$		176.41–0.07 *$T$		
${{c}_{p}}_{L}$	Specific heat	1413.50–0.01 *$T$	J/kg/K	
${{c}_{p}}_{\alpha }$		862.70 + 0.52 *$T$		
${\rho }_{L}$	Density	1834.74–0.26 *$T$	kg/m^3^	
${\rho }_{\alpha }$		1796.98–0.16*$T$		
$LT$	Latent heat	357,951	J/kg	[56]
$h$	Heat transfer coefficient	600	W/m^2^/K	[57]

### Mean-field modelling

The chemical potentials and diffusion coefficients in the SFFK mean-field model were obtained from the magnesium alloy database in Thermo-Calc. The compositional inputs for the SFFK mean-field model were set based on the segregation predictions from the Scheil model. The percentage of segregated compositions was approximated as the difference between the fraction of solids at the ternary eutectic point and at full solidification.

During rapid solidification, ZX10 and ZX70 generate a significant quantity of matrix phases as the dominant phases at their nominal compositions, leading to the formation of second phases with precipitation radius of less than 1 nm, which falls outside the predictive range of this model. Consequently, the SFFK mean-field model in this study focuses on the generation of second phases during the cooling of solid with segregated composition. The model calculates the volume fraction of second-phase precipitation in conjunction with the percentage of segregated compositions.

The mean-field coarsening predictions are highly sensitive to the values used to define the interfacial energies, which can be obtained for each precipitate in matrix from the database in the Thermo-Calc software and are given in Table 3. The continuity equations, particle growth rates, and nucleation rates were normalised and reformulated following the approach described by Anderson et al*.* [34, 58], and the advection equations are solved using the finite difference method. The nucleation of particles is carried out following the method of Jou et al. and the size change of the stable nucleus is described using a distribution function with a finer discretisation of the particle radius.

**Table 3:** Interfacial energy of precipitates in Mg–Zn–Ca Mg system rich corner

Precipitates	Interfacial energy	Units	References
Mg_2_Ca	0.06	J/m^2^	Thermo-Calc
MgZn	0.05		
Ca_2_Mg_6_Zn_3_	0.05		

The nucleation site fraction was determined by the dislocation density within grains, as outlined by Anderson et al*.* [35]. Grain boundary dislocations, which typically exhibit higher densities compared to the grain interior, act as nucleation sites for grain boundary precipitates. This relationship allows for the definition of the nucleation site fraction range within grains and facilitates the reverse estimation of precipitate segregation at various grain locations. When the nucleation site fraction is 1, precipitation approximates homogeneous nucleation, whereas lower nucleation site fraction values indicate a shift towards heterogeneous nucleation. In this study, the nucleation site fraction range is assumed to be between ${10}^{-5}$ and ${10}^{-15}$, decreasing by orders of magnitude. The effects of different combinations of nucleation site fraction ranges and cooling times on the precipitation behaviour of second phases are investigated.

From Eq. (16), it can be observed that the concentration of nuclei, ${N}_{0}$ is influenced by the nucleation site fraction, $\eta$. For homogeneous nucleation, $\eta$ can be approximated as 1, while for heterogeneous nucleation, $\eta$ can be estimated based on the dislocation density. Anderson et al*.* [35] derived an equation describing the relationship between the nucleation site fraction $\eta$ and dislocation density $\rho$, where $b$ is the Burgers vector. Since the measurement of dislocation density in each part of the sample is complicated, an estimate of nucleation site fraction is required.

24 $$\eta ={b}^{2}\rho$$

## Results

### Microstructure of as-cast ZX10 and ZX70 alloys

**Figure 7:**
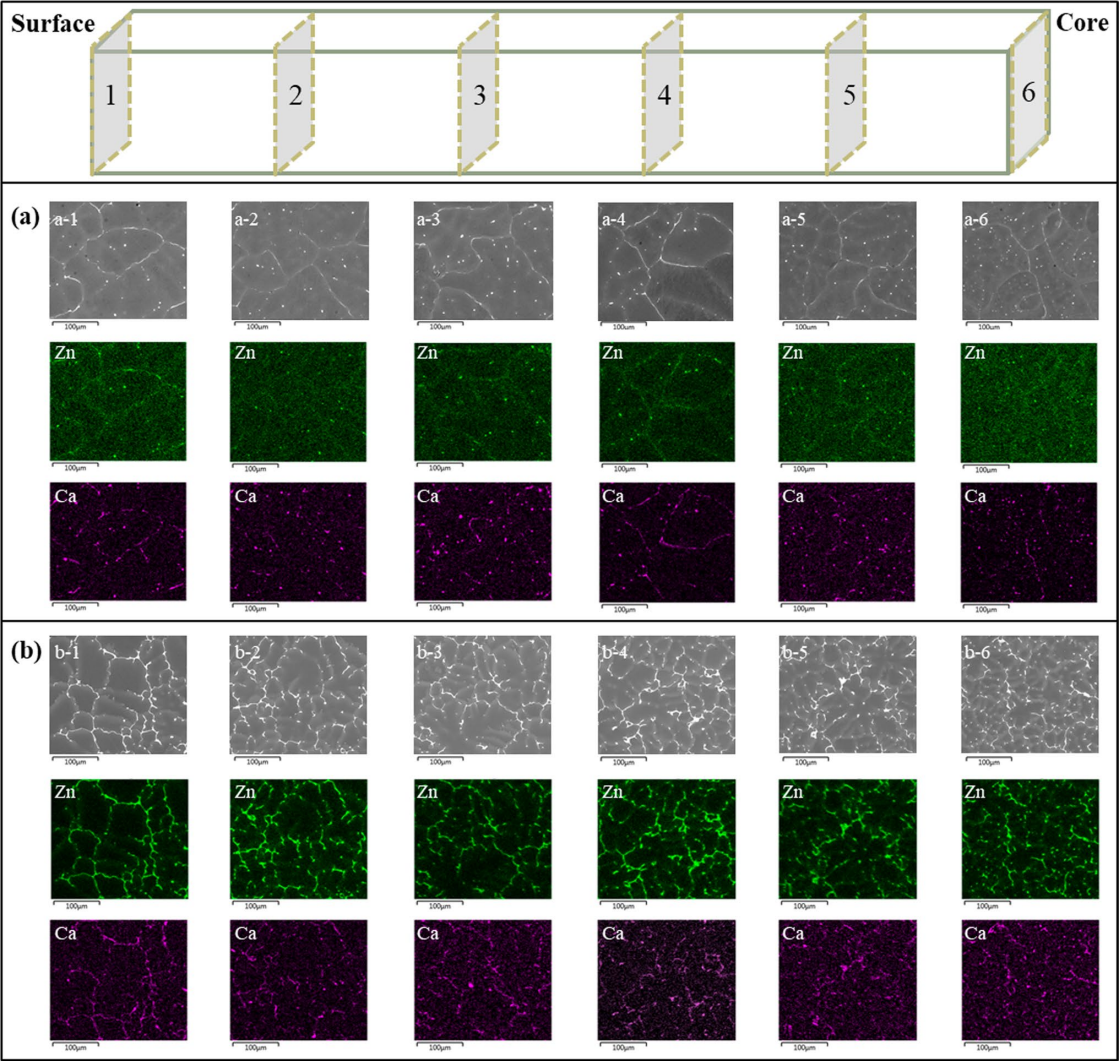
SEM and EDS map results of as-cast **a** ZX10 and **b** ZX70 alloys from surface (1) to core (6)

The microstructure of the ZX10 and ZX70 alloys in the as-cast condition is shown in Fig. 7. From the SEM images and EDS element distribution maps in Fig. 7a, significant Ca segregation is observed at the grain boundaries of the ZX10 alloy. While Zn segregation is also present at the grain boundaries, it is less pronounced compared to Ca, and the distribution of Zn appears relatively uniform within the Mg matrix, especially in regions closer to the centre of the cast sample (Fig. 7a-6). This phenomenon can be attributed to the gradual decrease in cooling rate from the surface to the centre of the cast sample. As the cooling rate slows, Zn segregation at the grain boundaries tends to decrease, whereas Ca segregation remains pronounced. Additionally, within the grains, a small number of spherical particles (~ 8 $\mu \text{m}$ in radius) containing both Zn and Ca are observed. These particles are likely Ca_2_Mg_6_Zn_3_ phases precipitated on micro-dendrites due to uneven solidification. A comparison of SEM images (Fig. 7a-1 to a-6) found that, as the cooling rate decreases, the density of particles within the grains gradually increases. This observation may suggest that the redistribution of Zn during solidification contributes to the increased particle density. The distribution of Ca and Zn elements is generally consistent; however, there are some particles and grain boundaries where Ca segregates independently. Therefore, it can be inferred that the segregated phases in ZX10 likely include both the Ca_2_Mg_6_Zn_3_ ​ phase and the Mg_2​_Ca phase.

The SEM images and EDS element distribution maps of ZX70 are shown in Fig. 7b. In ZX70, the distributions of Ca and Zn elements are generally consistent, with both segregating at the grain boundaries. However, compared to ZX10, the degree of Zn segregation at the grain boundaries in ZX70 is significantly higher, which can be attributed to the higher Zn content in the ZX70 alloy. The comparison of SEM images (Fig. 7b-1 to b-6) found that as the cooling rate decreases, the density of second phases in ZX70 gradually increases. Unlike the spherical precipitates formed in ZX10, ZX70 forms Zn-rich intermetallic compounds. Therefore, it can be inferred that in addition to the Ca_2_Mg_6_Zn_3_ ​ phase, ZX70 may contain a second phase that is different from the Mg_2​_Ca phase potentially present in ZX10. This second phase is preliminarily identified as MgZn.

### Scheil solidification

**Figure 8:**
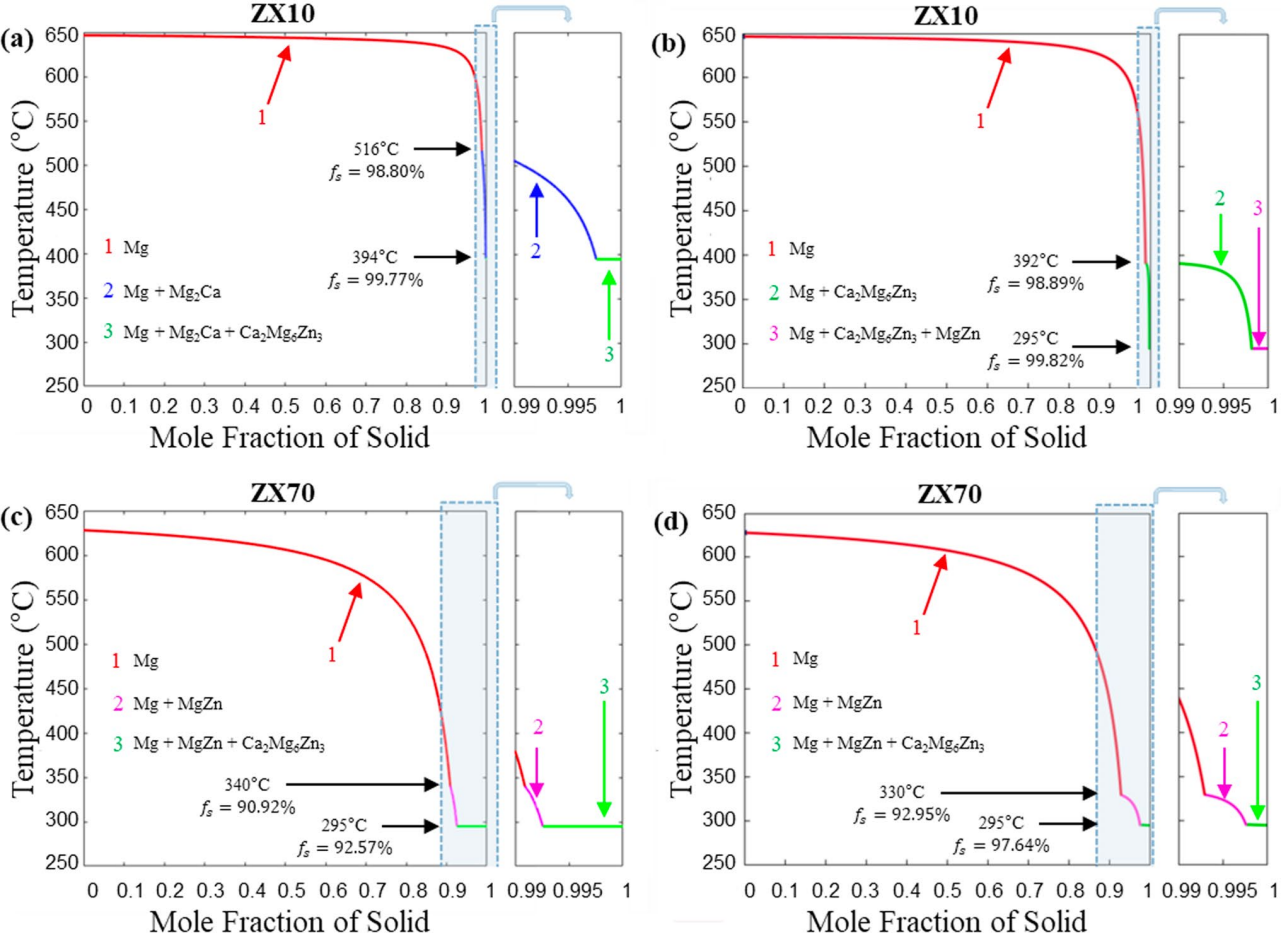
Scheil solidification curve of **a**, **b** ZX10 and **c**, **d** ZX70 alloy. **a**, **c** is generated from the liquidus-minimising Scheil in this study, and **b**, **d** is generated from Thermo-Calc Scheil calculator

Figure 8 illustrates the solidification paths of ZX10 and ZX70 alloys, comparing the results generated by the liquidus-minimising Scheil model developed in this study (Fig. 8a, c) with those from the Scheil model in Thermo-Calc software (Fig. 8b, d). The comparison reveals significant differences in the types, amounts, and temperatures of phases formed in ZX10 between the two models. In contrast, the types of phases formed in ZX70 are consistent between the models, with only minor differences observed in the amounts and temperatures.

Figure 8a, b illustrates the solidification paths of the ZX10 alloy predicted by liquidus-minimising Scheil model and Thermo-Calc software Scheil model, respectively. It can be observed that solidification begins at approximately 647 °C with the formation of the primary Mg matrix phase. In Fig. 8a, the Mg_2​_Ca phase starts forming alongside the primary Mg phase at approximately 516 °C and reaches the ternary eutectic point at 394 °C, where the Mg matrix phase, Mg_2​_Ca phase, and Ca_2_Mg_6_Zn_3_ phase solidify simultaneously. This result contrasts significantly with the findings shown in Fig. 8b. In Fig. 8b, the Ca_2_Mg_6_Zn_3_ ​ phase begins forming alongside the primary Mg phase at approximately 392 °C and reaches the ternary eutectic point at 295 °C, where the Mg matrix phase, Ca_2_Mg_6_Zn_3_ ​ phase, and MgZn phase solidify simultaneously. The large difference in eutectic temperatures (394 °C vs. 295 °C) arises primarily from how each model treats solute redistribution. These differences significantly impact phase sequence predictions and final microstructure, especially in ZX10 where small composition shifts dictate whether Mg_2_Ca or MgZn becomes dominant.

Figure 8c, 8 presents the solidification paths of the ZX70 alloy. In comparison, the differences between the liquidus-minimising Scheil model and the Thermo-Calc software Scheil model are relatively minor. Both models indicate that solidification begins at approximately 628 °C with the formation of the primary Mg matrix phase. Around 330–340 °C, the MgZn phase forms alongside the primary Mg phase, and the ternary eutectic point is reached at 295 °C, where the Mg matrix phase, MgZn phase, and Ca_2_Mg_6_Zn_3_​ phase solidify simultaneously. Although the overall phase types are similar, these differences in temperature and solid fraction at various solidification stages can be observed between the two models, factors that ultimately affect microstructure and property development. This is particularly important when tailoring solidification paths for alloy design, as in the optimisation of Mg–Zn–Ca systems.

Based on the SEM–EDS results from Fig. 7a, it is evident that the presence of the Mg_2​_Ca phase in the ZX10 alloy is more likely than the MgZn phase. To explore the significant differences observed between Fig. 8a, b, the potential effects of solute redistribution on local chemical equilibrium were considered. An equilibrium phase diagram for the ZX10 alloy (Mg–0.8Zn–0.2Ca, wt.%) with compositional fluctuations within a certain range was constructed. The selected composition range was derived from local compositions measured by SEM–EDS in Table 1.

**Figure 9:**
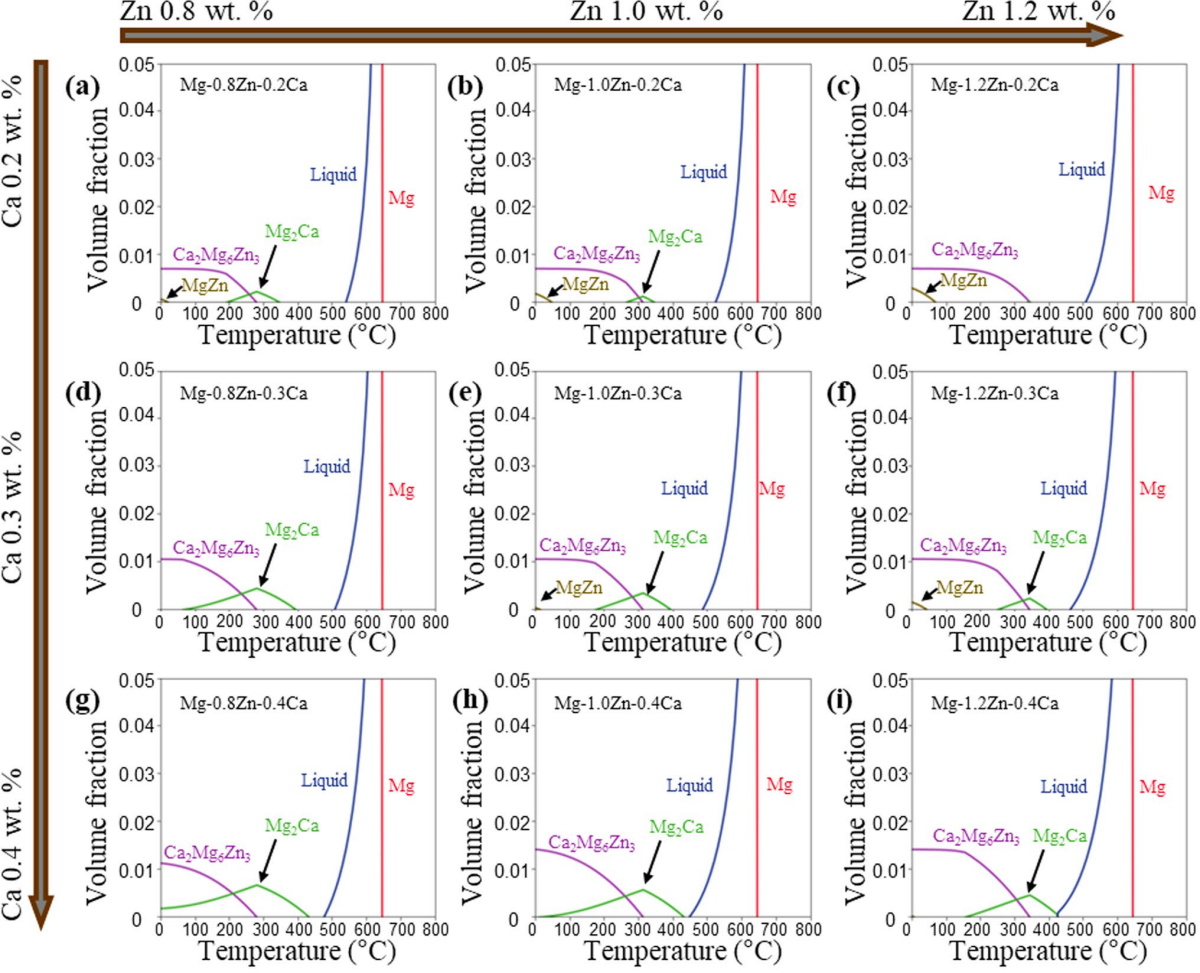
Equilibrium phase diagram of Mg–xZn–yCa (wt%) alloys, *x* = 0.8, 1.0, 1.2, and *y* = 0.2, 0.3, 0.4

As shown in Fig. 9, the equilibrium phase diagram shows that the formation of Mg_2​_Ca and MgZn phases is highly sensitive to compositional variations. Compared to the nominal composition of ZX10 (Mg–0.8Zn–0.2Ca, wt.%), an increase of 0.4 wt.% in Zn content may prevent the formation of Mg_2​_Ca during equilibrium solidification. Conversely, Ca segregation has an even more pronounced effect. An increase of 0.1–0.2 wt.% in Ca content can suppress the formation of MgZn while significantly increasing the amount of Mg_2​_Ca at lower temperatures.

These findings suggest that the nominal composition of the ZX10 alloy lies at the critical boundary for the formation of Mg_2​_Ca and MgZn phases. The compositional segregation caused by non-equilibrium solidification during rapid cooling, amplifies the differences in phase formation. Observing the liquidus-minimising Scheil solidification path of ZX10 (Supplementary Materials S1), Ca segregates first during the initial solidification stages, further increasing compositional differences and leading to Mg_2​_Ca as the initial second phase to form. In contrast, for ZX70 (Supplementary Materials S2), Zn segregates first, causing MgZn to become the initial second phase. Notably, differences in compositional segregation result in distinct solidification paths, which in turn lead to variations in the sequence, temperature, and solid fraction of second-phase formation. In the Mg–Zn–Ca system magnesium-rich corner, these differing solidification paths also point to different ternary eutectic points. The real-time phase fractions along the liquidus-minimising Scheil solidification paths of ZX10 and ZX70 are presented in Supplementary Materials S3 and S4. The phase fractions during solidification, collected from the TCMG6 database, are listed in Table 4.

**Table 4:** Phase transition during liquidus-minimising Scheil solidification for both ZX10 (Fig. 8a) and ZX70 (Fig. 8c) alloys

Alloy	Temperature	Phase transition	Mole fraction of Solid ${f}_{s}$
ZX10	647 °C < $T$ < 516 °C	$L\to 100\% \alpha (\text{Mg})$	0% < ${f}_{s}$< 98.80%
	516 °C < $T$ < 394 °C	$L\to 64.5\% \alpha (\text{Mg}) + 35.5\% {\text{Mg}}_{2}\text{Ca}$	98.80% < ${f}_{s}$< 99.77%
	394 °C	$L\to 35.9\% \alpha -\text{Mg} + 62.3\% {\text{Ca}}_{2}{\text{Mg}}_{6}\text{ Z}{\text{n}}_{3} + 1.8\% {\text{Mg}}_{2}\text{Ca}$	99.77% < ${f}_{s}$< 1
ZX70	628 °C < $T$ < 340 °C	$L\to 100\% \alpha (Mg)$	0% < ${f}_{s}$< 90.92%
	340 °C < $T$ < 295 °C	$L\to 52.4\% \alpha (\text{Mg}) + 47.6\% \text{MgZn}$	90.92% < ${f}_{s}$< 92.57%
	295 °C	$L\to 28.6\% \alpha (\text{Mg}) + 33.5\% {\text{Ca}}_{2}{\text{Mg}}_{6}\text{ Z}{\text{n}}_{3}+ 37.9\% \text{MgZn}$	92.57% < ${f}_{s}$< 1

Table 5 presents the final phase fractions in the solid state predicted by the liquidus-minimising Scheil model for ZX10 and ZX70. These results reflect the cumulative outcome of solidification up to the eutectic point and represent the expected volume proportions of each phase at room temperature, assuming negligible solid-state transformation thereafter.

**Table 5:** Final mole fractions of each solid phase predicted by the liquidus-minimising Scheil model

Alloy	Phase	Mole fraction of phase in Solid
ZX10	$\alpha -\text{Mg}$	0.99505
	${\text{Mg}}_{2}\text{Ca}$	0.00349
	${\text{Ca}}_{2}{\text{Mg}}_{6}{\text{Zn}}_{3}$	0.00145
ZX70	$\alpha -\text{Mg}$	0.93909
	$\text{MgZn}$	0.03601
	${\text{Ca}}_{2}{\text{Mg}}_{6}{\text{Zn}}_{3}$	0.02490

### Mean-field SFFK results of second-phase precipitation

The Scheil model is used to simulate the solidification process from the liquid to the solid phase, while the mean-field SFFK model is applied to simulate the cooling process of the solid phase from the liquid–solid transition temperature to room temperature There is uncertainty to the exact nucleation site fraction for the second-phase precipitates, in addition to the exact cooling rate after solidification. In this study, the predicted range of the nucleation site fraction for second-phase particles is set between ${10}^{-5}$ and ${10}^{-15}$, decreasing in orders of magnitude. Cooling times range from 5 to 120 min, with increments of 5 min. Colour gradients indicate the predicted volume fraction of specific second phases.

**Figure 10:**
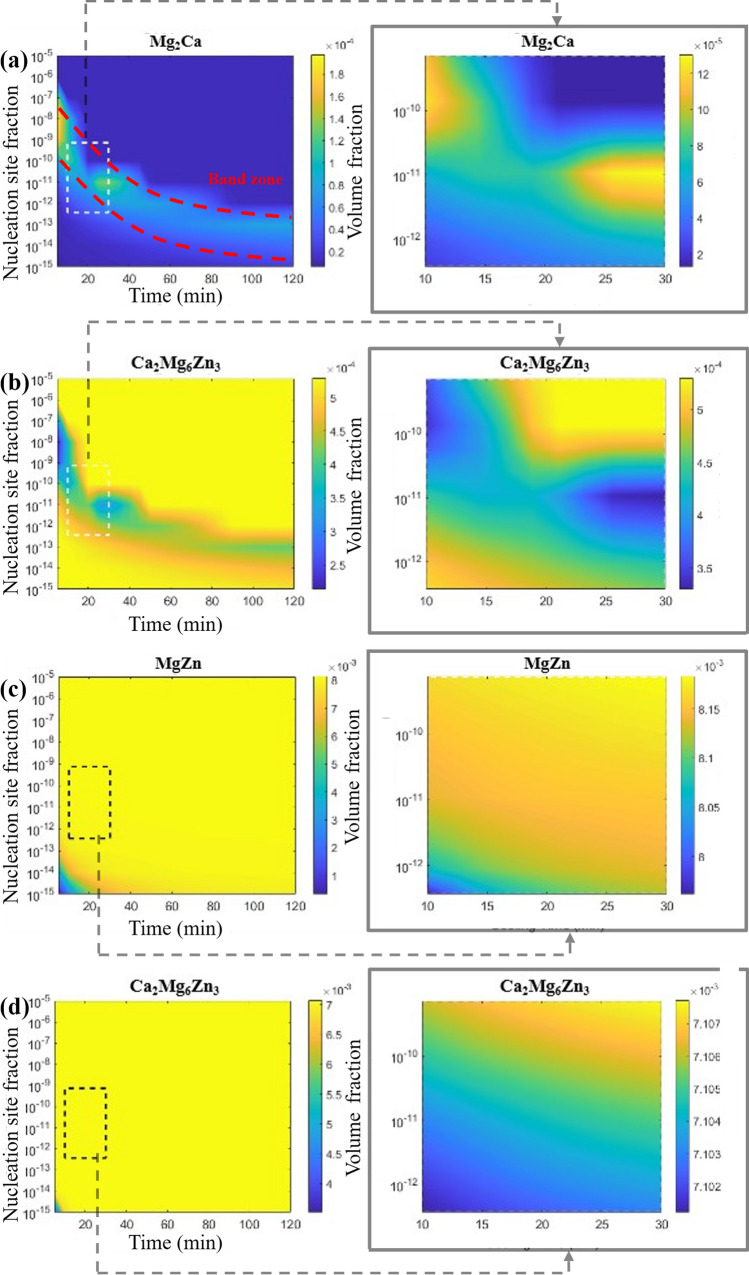
Particles volume fraction with variation of nucleation site fraction and cooling time in ZX10 (**a**, **b**) and ZX70 (**c**, **d**) alloys; **a** is Mg_2_Ca phase, **b** is Ca_2_Mg_6_Zn_3_ phase in ZX10; **c** is MgZn phase, **d** is Ca_2_Mg_6_Zn_3_ phase in ZX70

Figure 10 illustrates the results of the mean-field SFFK model, showing the evolution of second-phase volume fractions in the Mg matrix of ZX10 and ZX70 alloys under varying nucleation site fractions and cooling times. Figure 10a illustrates the formation of second phases in the ZX10 alloy. The Mg_2_​Ca phase is observed to form at a nucleation site fraction of ${10}^{-5}$ under the 5 min of cooling time, with its maximum volume fraction reaching approximately $2\times {10}^{-4}$. Notably, a band zone is present in Fig. 10a, where the volume fraction of Mg_2_​Ca is significantly higher in the central region compared to other areas. A comparison between Fig. 10a, b shows a competitive relationship, where increased formation of Mg_2_​Ca correlates with a decrease in Ca_2_Mg_6_Zn_3_​​ volume fraction. In regions where Mg_2_​Ca forms in higher quantities, the volume fraction of Ca_2_Mg_6_Zn_3_​​ decreases correspondingly. In areas where less Mg_2_​Ca formed, the volume fraction of Ca_2_Mg_6_Zn_3_​​ remains approximately $5\times {10}^{-4}$. Additionally, by analysing the magnified areas of Fig. 10a, b, it can be observed that the reduction in Ca_2_Mg_6_Zn_3_​​ volume fraction corresponds approximately to the increase in Mg_2_​Ca volume fraction, further confirming their competitive relationship. Interestingly, a small peak in the volume fraction of Mg_2_​Ca approximately $1.3\times {10}^{-4}$ is noted at 30 min with a nucleation site fraction of ${10}^{-11}$, after which the volume fraction of Mg_2_​Ca gradually decreases.

Figure 10c, d shows the formation of second phases in the ZX70 alloy. Except for the region near the lower-left corner, the formation of MgZn and Ca_2_Mg_6_Zn_3_​ phases is relatively uniform. Upon comparing the magnified areas of Fig. 10c, d, it is evident that both MgZn and Ca_2_Mg_6_Zn_3_​ phases tend to form under higher nucleation site fractions and longer cooling times, although the increase in their volume fractions is not particularly significant. The volume fraction of MgZn stabilises at approximately $8\times {10}^{-3}$, while that of Ca_2_Mg_6_Zn_3_​ stabilises at approximately $7\times {10}^{-4}$. Unlike in ZX10, no apparent competitive relationship between the formation of MgZn and Ca_2_Mg_6_Zn_3_​ phases is observed in the Mg matrix of ZX70. These results suggest that in ZX10, the formation of Mg_2_​Ca and Ca_2_Mg_6_Zn_3_​ phases is strongly interdependent, with significant competition affecting their respective volume fractions. In contrast, the formation of MgZn and Ca_2_Mg_6_Zn_3_​ phases in ZX70 appears to occur more uniformly and independently, without notable interactions between the two phases.

**Figure 11:**
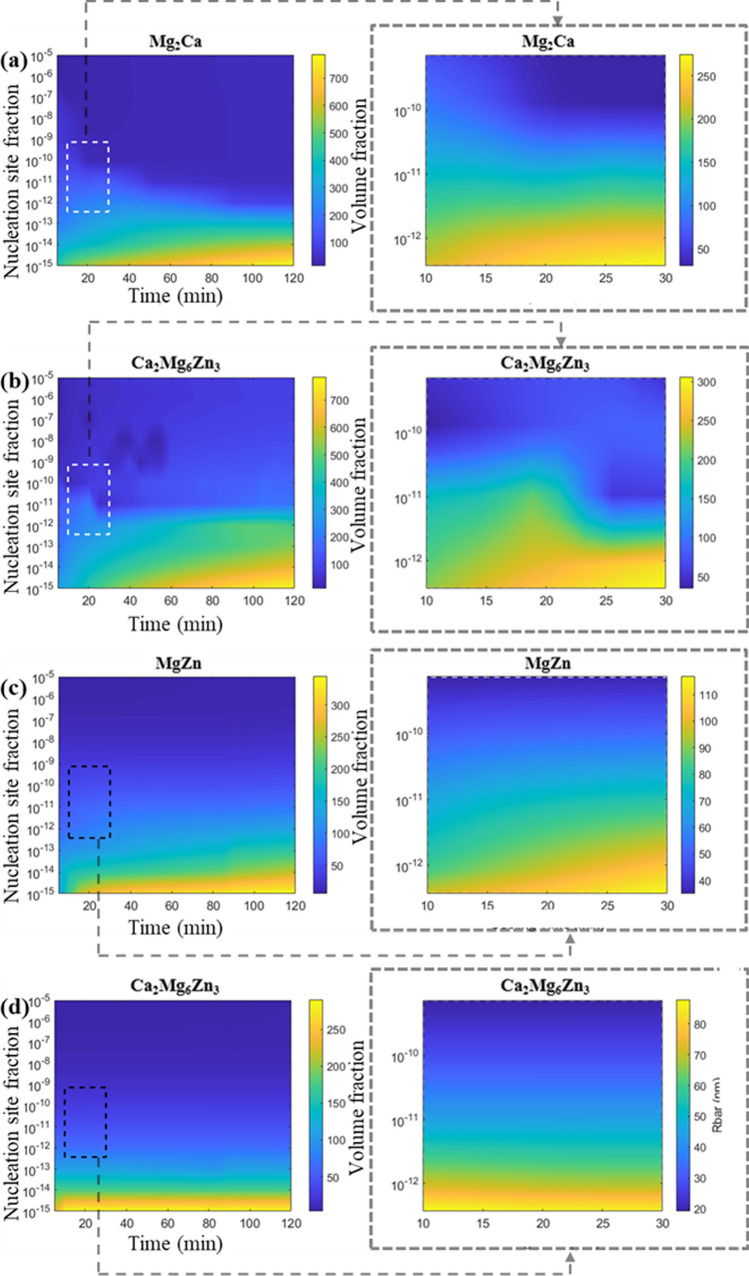
Particles mean radius with variation of nucleation site fraction and cooling time in ZX10 (**a**, **b**) and ZX70 (**c**, **d**) alloys; **a** is Mg_2_Ca phase, **b** is Ca_2_Mg_6_Zn_3_ phase in ZX10; **c** is MgZn phase, **d** is Ca_2_Mg_6_Zn_3_ phase in ZX70

Figure 11 presents the mean-field SFFK model results for the mean radius of second-phase particles during the cooling process in a Mg matrix under varying nucleation site fractions and cooling times. The results indicate that all second-phase particles tend to form at lower nucleation site fractions, with their mean radius increasing as the cooling duration extends. However, the particles formed in the ZX10 alloy are larger than those in the ZX70 alloy. At a nucleation site fraction of ${10}^{-15}$, the maximum mean radius of particles in ZX10 and ZX70 are approximately 800 nm and 300 nm, respectively.

In the ZX10 alloy, as shown in Fig. 11a, b, when the nucleation site fraction is higher than the band zone identified in Fig. 10, the mean radius of Mg_2_​Ca and Ca_2_Mg_6_Zn_3_​ particles do not significantly increase with extended cooling time. Under these conditions, the mean radius of Mg_2_​Ca particles remains between 10 and 50 nm, while that of Ca_2_Mg_6_Zn_3_​ particles is between 100 and 200 nm. However, when the nucleation site fraction is lower than the band zone, the mean radius of both particles increases substantially with decreasing nucleation site fraction and longer cooling durations. At a nucleation site fraction of ${10}^{-15}$ and a cooling duration of 120 min, the mean radius of both Mg_2_​Ca and Ca_2_Mg_6_Zn_3_​ particles reach approximately 800 nm.

In the ZX70 alloy, as showing in Fig. 11c, d, the particle size of MgZn and Ca_2_Mg_6_Zn_3_​ also increases with decreasing nucleation site fraction. At a nucleation site fraction of ${10}^{-15}$ and a cooling duration of 120 min, the maximum mean radius of MgZn particles reaches approximately 350 nm, while that of Ca_2_Mg_6_Zn_3_​ particles is approximately 300 nm. However, when comparing the mean radius of MgZn and Ca_2_Mg_6_Zn_3_​, MgZn particles exhibit a gradual increase in size with extended cooling time, whereas the mean radius of Ca_2_Mg_6_Zn_3_​ particles remains relatively unchanged.

## Discussion

### Quantitative analysis

To evaluate whether the second phases originate from the liquid or the magnesium matrix, quantitative analysis of the density and size of second phases in Fig. 7 was performed using ImageJ software. The results were compared with the volume fraction predicted by the Scheil model and the mean-field SFFK model, which assumes a nucleation site fraction of ${10}^{-15}$, averaged over all temperature range in Fig. 7. The results are summarised in Tables 6 and 7.

**Table 6:** Quantitative analysis of the area fraction of second-phase particles, in comparison with the volume fraction predicted by the mean-field SFFK model and the Scheil model, was conducted for both ZX10 and ZX70 alloys

Location	*1*		*2*	*3*	*4*	*5*	*6*
Second-phase particles area fraction ${A}_{f}$ from Fig. 7							
ZX10	$8.64\times {10}^{-3}$		$8.36\times {10}^{-3}$	$9.69\times {10}^{-3}$	$9.24\times {10}^{-3}$	$8.01\times {10}^{-3}$	$9.22\times {10}^{-3}$
Mean area fraction ${A}_{f}$	$8.86\times {10}^{-3}\pm 6.3\times {10}^{-4}$						
ZX70	$4.60\times {10}^{-2}$		$4.95\times {10}^{-2}$	$4.71\times {10}^{-2}$	$6.70\times {10}^{-2}$	$5.48\times {10}^{-2}$	$4.11\times {10}^{-2}$
Mean area fraction ${A}_{f}$	$5.09\times {10}^{-2}\pm 9.1\times {10}^{-3}$						
Second-phase particles mean volume fraction ${V}_{f}$ from mean-field model							
ZX10	$5.41\times {10}^{-4}\pm 1.92\times {10}^{-6}$						
ZX70	$1.38\times {10}^{-2}\pm 2.55\times {10}^{-3}$						
Second-phase particles mean fraction ${V}_{f}$ from Scheil model							
ZX10	$5.61\times {10}^{-3}$						
ZX70	$5.93\times {10}^{-2}$

**Table 7:** Quantitative analysis of the mean radius of second-phase particles, in comparison with the mean radius predicted by the mean-field SFFK model, was conducted for both ZX10 and ZX70 alloys

Location	1	2	3	4	5	6
Second-phase particles mean radius $\overline{R }$ (μm) from Fig. 7						
ZX10	7.14	6.24	7.99	7.76	5.13	3.91
Average $\overline{R }$	$6.36\pm 1.60$					
ZX70	19.00	11.80	14.29	16.39	19.48	11.40
Average $\overline{R }$	$15.39\pm 3.49$					
Second-phase particles average mean radius $\overline{R }$ (μm) from mean-field model						
ZX10	$0.64\pm 0.12$					
ZX70	$0.30\pm 0.03$					

Table 6 shows the area fraction of second-phase particles at each location in Fig. 7 for the ZX10 and ZX70 alloys, along with the mean area fraction across all locations. For ZX10 and ZX70, the Scheil model predicts second-phase fractions ($5.61\times {10}^{-3}$ and $5.93\times {10}^{-2}$, respectively) that closely match the measured area fractions ($8.86\times {10}^{-3}$ and $5.09\times {10}^{-2}$), while the SFFK model significantly underestimates these values ($5.41\times {10}^{-4}$ and $1.38\times {10}^{-2}$, respectively). Although area fractions derived from 2D SEM images are not strictly equivalent to volume fractions, the magnitude of discrepancy between the two models is sufficient to suggest that most second-phase particles formed directly from the liquid during solidification rather than through subsequent solid-state precipitation.

In addition, Table 7 presents the corresponding particle sizes. The mean radii measured from SEM images are 6.36 μm (ZX10) and 15.39 μm (ZX70), compared to SFFK model predictions of 0.64 μm and 0.30 μm. These large differences in particle size further reinforce the conclusion that the observed second phases are not the result of diffusion-limited coarsening from a supersaturated Mg matrix, but rather originate predominantly from primary solidification.

### Second-phase formation in Scheil solidification model

Differences in the partition coefficient $k$ and the numerical methods employed in Scheil calculations significantly impact the accuracy of predicted solidification behaviour, particularly in multicomponent alloy systems [40, 59, 60]. The partition coefficient $k$ determines solute redistribution during solidification and plays a critical role in establishing phase stability, formation temperature, and phase evolution sequence. Variations in the partition coefficients used by the liquidus-minimising Scheil model and the Thermo-Calc Scheil model can result in notable differences in predictions of phase stability and solidification sequences.

The choice of numerical methods further amplifies these discrepancies, as it governs how the precision with which local phase equilibrium is captured. The liquidus-minimising Scheil model uses a gradient-minimising approach with a compositional gradient input step size of 0.1 at%, ensuring that the solidification path follows the fastest decreases along the liquidus surface. This approach provides a finer representation of compositional segregation and phase transformations, particularly in systems where minor compositional changes significantly affect phase equilibrium. In contrast, the Thermo-Calc Scheil model employs a more generalised algorithm that simplifies the solidification path using a temperature gradient input step size of 1 Kelvin. While sufficient for many applications, this method may underestimate or overestimate critical phase transformation temperatures and solid fractions. For instance, as observed in the ZX70 alloy, there are slight deviations in the solidification predictions between the two models. In the ZX10 alloy, the Liquidus-minimising model predicts the formation of Mg_2_​Ca at 516 °C, whereas the Thermo-Calc Scheil model indicates a much lower formation temperature.

The differences observed between the Liquidus-minimising and Thermo-Calc Scheil models have significant implications for Mg–Zn–Ca alloy optimisation. For compositions sensitive to segregation, such as the ZX10 alloy, even minor discrepancies in $k$ values or numerical modelling can lead to substantial variations in phase distribution and mechanical properties. The liquidus-minimising model’s ability to capture subtle changes in phase equilibrium and segregation paths provides a more detailed understanding of solidification behaviour. In contrast, while the Thermo-Calc Scheil model is convenient for preliminary analyses, it may require enhancements to address the specific demands of complex ternary systems.

### Solidification segregation and composition map

**Figure 12:**
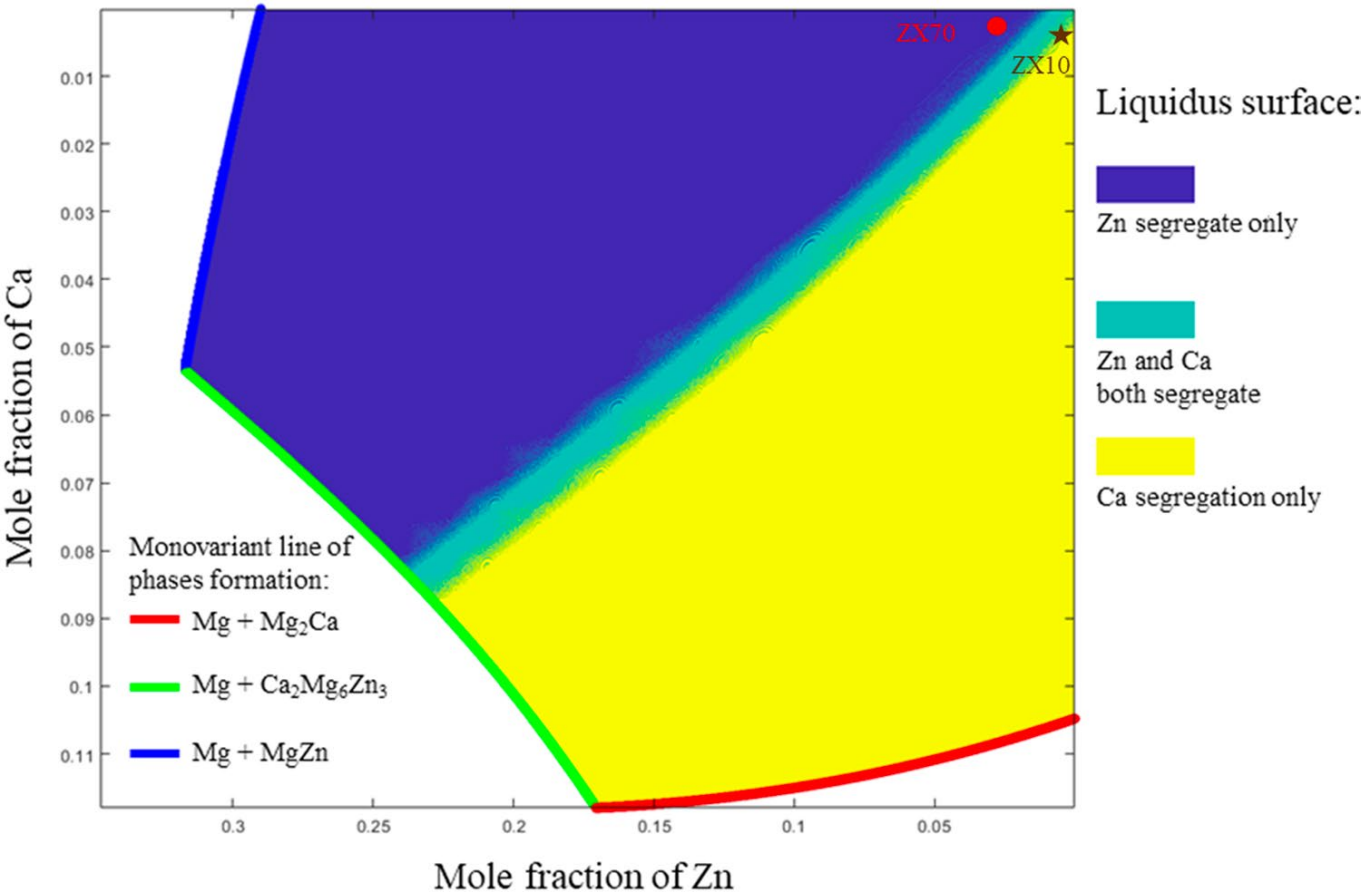
Composition map of Mg–Zn–Ca alloys solidification segregation pathway

The results of the liquidus-minimising Scheil model indicate that ZX10 and ZX70 alloys exhibit distinct solidification paths, with the composition of ZX10 being closer to the region sensitive to changes in segregation paths compared to ZX70. The compositional diagram in Fig. 12 illustrates the areas within the Mg–Zn–Ca magnesium-rich corner that are prone to specific elemental segregation. As shown in Fig. 12, ZX10 is located near the region where both Zn and Ca segregate simultaneously and where Ca segregates independently. This positioning leads to pronounced Ca segregation as solidification progresses. In contrast, ZX70 is situated in the region where Zn segregates independently, making the alloy less sensitive to compositional heterogeneity during non-equilibrium solidification. Considering that the Ca_2_Mg_6_Zn_3_ ​ phase can enhance the mechanical properties of alloys [26], the compositional diagram in Fig. 12 also provides valuable guidance for alloy design. This figure identifies compositional regions where Zn and Ca are likely to segregate either independently or simultaneously, thus influencing which intermetallic phase is more likely to form. Alloys located near the Ca-dominant region (e.g. ZX10) favour Mg_2_Ca formation, while those closer to the Zn-dominant corner (e.g. ZX70) are more prone to MgZn formation. Compositions near the co-segregation boundary are ideal for promoting fine Ca_2_Mg_6_Zn_3_ precipitation. Therefore, this map can serve as a design tool for selecting Mg–Zn–Ca compositions that optimise the formation of desired intermetallic phases.

#### Precipitation sequences of Mg–Zn precipitates

**Figure 13:**
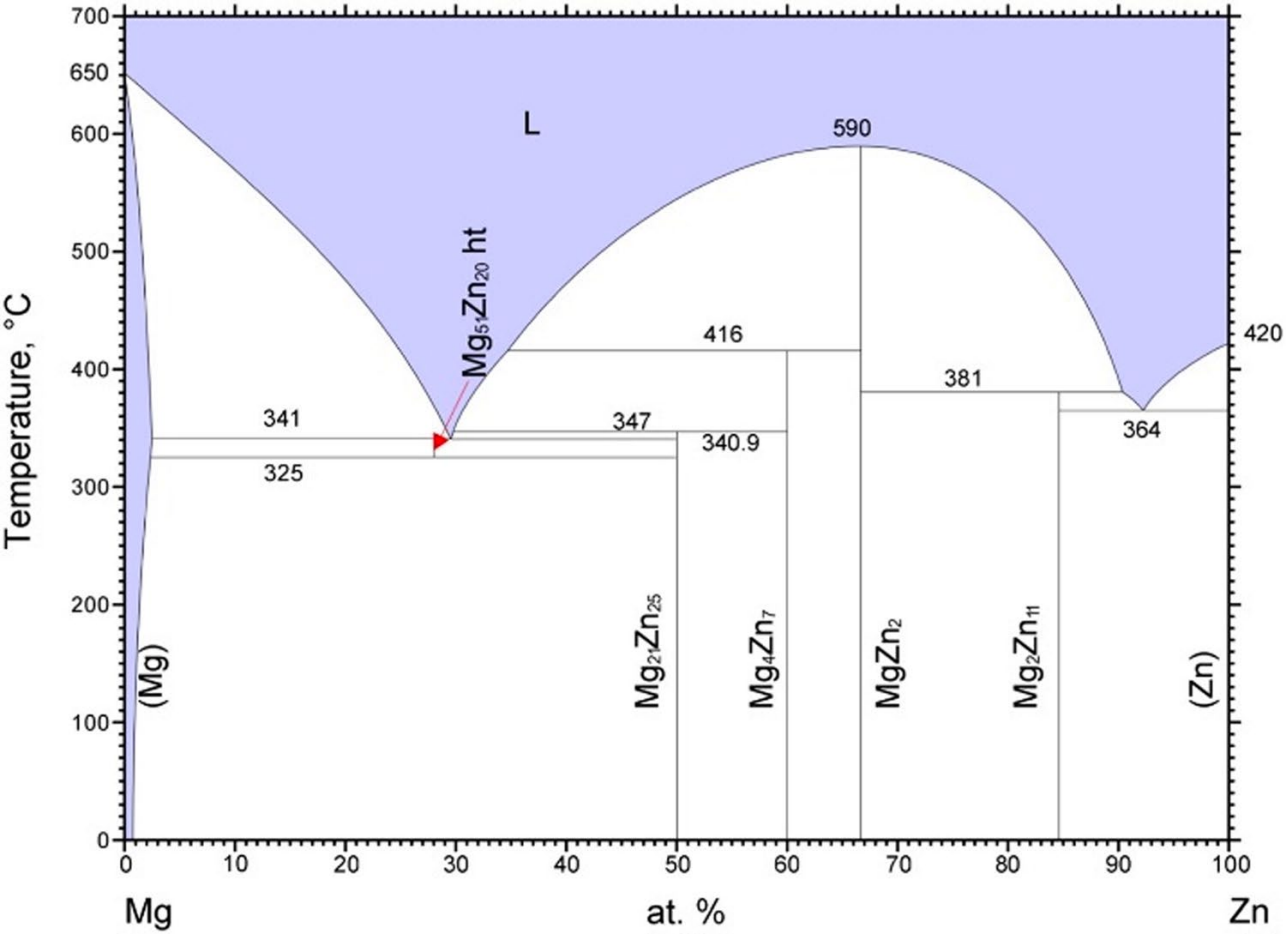
Phase diagram of Mg–Zn system [16]

In Mg–Zn–Ca alloy system, the precipitation sequences in Mg–Zn binary side are more complex than that of the Mg–Ca side. Figure 13 shows the equilibrium phases in Mg–Zn binary system, in which ${\text{Mg}}_{21}{\text{Zn}}_{25}$ also known as MgZn [16]. From Fig. 13, we can see that at the Mg-enriched corner, stable ${\text{Mg}}_{51}{\text{Zn}}_{20}$ phase will form between temperature 325 °C and 341 °C, which is going to form at the eutectic point with $30 \text{at}.\%$ of Zn before MgZn phase during the cooling process [61]. In addition, different metastable binary phases are formed during the precipitation process in Mg-Zn alloys, and the generally accepted precipitation sequence is $SSSS\to GP zone\to {\beta }_{1}{\prime}\to {\beta }_{2}{\prime}\to \beta$, in which $\beta$ is the equilibrium phase MgZn [62, 63]. The GP zone has been described as a coherent nanoscale precipitate of several atomic layers on certain crystalline surfaces of the Mg matrix; however, it is still not directly observable because of its small size and coherence with the matrix. Bhattacharjee et al. reported the formation of GP zone in Mg–2.4 Zn at.% alloy by three-dimensional atom probe (3DAP) and observed the existence of Zn clusters [64]. ${\beta }_{1}{\prime}$ usually has a rod-like morphology vertical to the basal plane of the Mg matrix and occurs in peak-aged samples, with the currently widely accepted composition being a mixture of ${\text{Mg}}_{4}{\text{Zn}}_{7}$ and ${\text{MgZn}}_{2}$ phases [65, 66]. ${\beta }_{2}{\prime}$ has a plate-like morphology form at basal plane of Mg matrix and occurs at over-aged samples, which has confirmed with composition as ${\text{MgZn}}_{2}$ [62, 67, 68]. In this study, only stable phases were considered in phase diagram calculations, as the TCMG6 thermodynamic database does not include metastable binary phases such as $\text{GP zone}, {\beta }_{1}^{\prime}, {\text{or}} \beta _{2}^{\prime}$. While these metastable precipitates are known to contribute to age hardening in Mg–Zn systems, their formation and transformation kinetics were not explicitly modelled here. For simplicity and thermodynamic consistency, we used MgZn to represent the aggregate behaviour of Mg–Zn binary precipitates. This approximation is sufficient for capturing segregation trends during solidification, which is the primary focus of this study.

## Conclusion

This study systematically explored the solidification and phase formation behaviour of the rapid cooling Mg–0.8 wt.% Zn–0.2 wt.% Ca (ZX10) and Mg–6.8 wt.% Zn–0.2 wt%. Ca (ZX70) alloys using a combination of the newly developed liquidus-minimising Scheil model and mean-field SFFK modelling approaches, integrated with CALPHAD thermodynamic simulations. The following conclusions may be drawn from the present study:

1. SEM–EDS analysis shows that both ZX10 and ZX70 alloys exhibit co-segregation of Ca and Zn at grain boundaries. However, ZX10 contains particles with Ca segregation alone, while ZX70 contains particles with Zn segregation alone. Based on this observation, it can be inferred that Mg_2_​Ca is likely to form in ZX10, whereas MgZn is expected to form in ZX70.
2. A comparison of the results from the Scheil model, the mean-field SFFK model, and the quantitative analysis of SEM images in Tables 6 and 7 shows that the second-phase volume fractions ${(V}_{f})$ predicted by the liquidus-minimising Scheil model ($5.61\times {10}^{-3}$ for ZX10 and $5.93\times {10}^{-2}$ for ZX70. respectively) are closer to the area fractions $({A}_{f})$ obtained from the quantitative analysis ($8.86\times {10}^{-3}$ for ZX10 and $5.09\times {10}^{-2}$ for ZX70, respectively). In contrast, the second-phase particle sizes $(\overline{R })$ predicted by the mean-field SFFK model ($0.64$ μm for ZX10 and $0.30$ μm for ZX70, respectively) are significantly smaller than the particle sizes observed in the analysis ($6.36$ μm for ZX10 and $15.39$ μm for ZX70, respectively). These findings suggest that second-phase particles are more likely to form directly from the liquid during solidification rather than precipitate from the supersaturated Mg matrix. Therefore, the newly developed liquidus-minimising Scheil model provides predictions that more accurately reflect the behaviour of rapidly solidified Mg–Zn–Ca alloys compared to the mean-field SFFK model. This supports the conclusion that hypothesis 1 is the dominant mechanism under the studied conditions, while hypothesis 2 plays a secondary role limited to minimal post-solidification coarsening.
3. The liquidus-minimising Scheil model developed in this study provides an accurate method for simulating segregation during non-equilibrium solidification, effectively capturing compositional gradient changes and predicting phase formation in both alloys. This approach is particularly effective for ternary alloys, where phase behaviour is highly sensitive to minor compositional variations. A comparison with the Scheil calculator in the commercial software Thermo-Calc using TCMG6 shows that while the Thermo-Calc model predicts similar solidification trends, it exhibits differences in the formation temperatures and sequence of second phases, especially in ZX10 alloys, which are highly sensitive to compositional changes during solidification. The results underscored the critical role of compositional control. ZX10 exhibited heightened sensitivity to minor compositional variations, leading to distinct solidification paths and phase distributions, while ZX70 showed more stable phase behaviour.
4. The findings highlight the critical role of precise compositional control and the use of advanced Scheil modelling to optimise the solidification pathways of Mg–Zn–Ca alloys. The differences in the solidification paths of ZX10 and ZX70 are attributed to their varying compositional sensitivities, particularly the segregation behaviour of Zn and Ca.

## Supplementary Information

Below is the link to the electronic supplementary material.

Supplementary file1 (MP4 2718 kb)

Supplementary file2 (MP4 3604 kb)

## Appendix: Derivation of Lever rule from binary system to ternary system

The initial solidification points at liquidus surface: ${\text{P}}_{0}\left[{w}_{a}\left(0\right), {w}_{b}\left(0\right), {w}_{c}\left(0\right), {T}_{0}\right]$

The solidification points $i$ at liquidus surface: ${\text{P}}_{i}\left[{w}_{a}\left(i\right), {w}_{b}\left(i\right), {w}_{c}\left(i\right), {T}_{i}\right]$

The final solidification points at liquidus surface: ${\text{P}}_{\text{end}}\left[{w}_{a}\left(end\right), {w}_{b}\left(end\right), {w}_{c}\left(end\right), {T}_{end}\right]$

25 $$\begin{aligned} f_{s} \left( i \right) & = \left| {\frac{{w_{b} \left( i \right) - w_{b} \left( 0 \right)}}{{w_{b} \left( i \right)}}} \right| \\ & w_{b} \left( 0 \right) < w_{b} \left( i \right) < w_{b} \left( {end} \right) \\ \end{aligned}$$

26 $$\begin{aligned} f_{L} \left( i \right) & = \frac{{w_{b} \left( 0 \right)}}{{w_{b} \left( i \right)}} \\ & w_{b} \left( 0 \right) < w_{b} \left( i \right) < w_{b} \left( {end} \right) \\ \end{aligned}$$

Expended from binary to ternary, apply Pythagorean theorem,

27 $$\begin{aligned} w_{b,c}^{2} & = w_{b}^{2} + w_{c}^{2} \\ & w_{b} \left( 0 \right) < w_{b} \left( i \right) < w_{b} \left( {end} \right) \\ & w_{c} \left( 0 \right) < w_{c} \left( i \right) < w_{c} \left( {end} \right){ } \\ \end{aligned}$$

From Eq. (25), we can know

28 $${f}_{s}^{2}\left(i\right)=\frac{{\left({w}_{b,c}\left(i\right)-{w}_{b,c}\left(0\right)\right)}^{2}}{{w}_{b,c}^{2}\left(i\right)}$$

Apply Eq. (27) in Eq. (28),

29 $${f}_{s}^{2}\left(i\right)=\frac{{w}_{b}^{2}\left(i\right)+{w}_{c}^{2}(i)+{w}_{b}^{2}\left(0\right)+{w}_{c}^{2}(0)-2\sqrt{\left({w}_{b}^{2}\left(i\right)+{w}_{c}^{2}\left(i\right)\right)\left({w}_{b}^{2}\left(0\right)+{w}_{c}^{2}\left(0\right)\right)}}{{w}_{b}^{2}\left(i\right)+{w}_{c}^{2}(i)}$$

From Eq. (26), we can know

30 $${f}_{L}\left(i\right)=\frac{{w}_{b}(0)}{{w}_{b}(i)}=\frac{{w}_{c}(0)}{{w}_{c}(i)}$$

Then,

31 $$\sqrt{\left({w}_{b}^{2}\left(i\right)+{w}_{c}^{2}\left(i\right)\right)\left({w}_{b}^{2}\left(0\right)+{w}_{c}^{2}\left(0\right)\right)}={w}_{b}^{2}\left(i\right){w}_{b}^{2}\left(0\right)+{w}_{c}^{2}\left(i\right){w}_{c}^{2}\left(0\right)$$

Apply Eq. (31) in Eq. (29),

32 $${f}_{s}^{2}\left(i\right)=\frac{{w}_{b}^{2}\left(i\right)+{w}_{c}^{2}(i)+{w}_{b}^{2}\left(0\right)+{w}_{c}^{2}(0)-2\left({w}_{b}^{2}\left(i\right){w}_{b}^{2}\left(0\right)+{w}_{c}^{2}\left(i\right){w}_{c}^{2}\left(0\right)\right)}{{w}_{b}^{2}\left(i\right)+{w}_{c}^{2}(i)}$$

Then,

33 $$\begin{aligned} f_{s} \left( i \right) & = \sqrt {\frac{{\left( {w_{b} \left( i \right) - w_{b} \left( 0 \right)} \right)^{2} + \left( {w_{c} \left( i \right) - w_{c} \left( 0 \right)} \right)^{2} }}{{w_{b} \left( i \right)^{2} + w_{c} \left( i \right)^{2} }}} \\ & w_{b} \left( 0 \right) < w_{b} \left( i \right) < w_{b} \left( {end} \right) \\ & w_{c} \left( 0 \right) < w_{c} \left( i \right) < w_{c} \left( {end} \right) \\ \end{aligned}$$
